# Mechanical Properties of Amide Functionalized CNT/NBR at Different Temperatures: A Molecular Dynamics Study

**DOI:** 10.3390/polym14071307

**Published:** 2022-03-24

**Authors:** Longcheng Ji, Lijia Chen, Li Lin, Shijie Wang

**Affiliations:** 1 School of Materials Science and Engineering, Shenyang University of Technology, Shenyang 110870, China; jilongcheng1@163.com (L.J.); linli-1969@126.com (L.L.); 2 School of Mechanical Engineering, Shenyang University of Technology, Shenyang 110870, China; wang_shijie@263.com

**Keywords:** molecular dynamics simulation, NBR, amide, mechanical properties, fractional free volume

## Abstract

A comprehensive study on the mechanical properties of a pure carbon nanotube (PCNT)/nitrile butadiene rubber (NBR) composite and an amide-functionalized carbon nanotube (CONH_2_–CNT)/nitrile butadiene rubber (NBR) composite was carried out using molecular dynamics (MDs) simulations at different temperatures. The effects of temperature on the mechanical properties, fractional free volume (FFV), MSD, dipole autocorrelation function, number of hydrogen bonds of PCNT composites, and functionalized CNT composites were analyzed and compared, and the pull-out behavior of the composites under different condition temperatures was simulated. The enhancement mechanism of the interface interaction between the functionalized carbon nanotubes and the NBR matrix was explained from an atomic point of view. The results show that, due to the existence of hydrogen bonds, higher interfacial binding energies were formed between PCNT and NBR, and FFVs and MSDs were restricted at each temperature, with the mechanical properties of the composites being improved by 5.02–25.93%.

## 1. Introduction

NBR is a random copolymer prepared by free-radical-initiated emulsion polymerization of two monomers, butadiene and acrylonitrile [1]. Due to the polar nitrile group having good oil resistance, heat resistance, and good physical and mechanical properties in their molecular structure, they are widely used in the automotive, aerospace, petroleum exploration, petrochemical, textile, wire and cable, printing, and food-packaging fields [2]. NBR is different from natural rubber or neoprene, in that it has a poor crystallization ability under tension, and therefore lacks its reinforcing effect [3]. Therefore, the strength of the pure NBR formula is very low, and the practical value is very small. The reinforcing phase must be used in the formulation of NBR, and its variety is closely related to the properties of NBR [4].

In addition to having particularly large tensile modulus and tensile strength, carbon nanotubes also have outstanding electrical conductivity and thermal conductivity [5], which are currently the best and most traditional reinforcing agents. Therefore, the simulation study of the relationship between carbon nanotubes and the mechanical properties of NBR through molecular dynamics is particularly important for the rubber-processing industry. According to recent research, amide has therefore been adopted to modify the CNTs to render high mechanical performance to CNT/NBR composites, since there were further numerous stable chemical modifications from amide linkages of the functionalized CNTs, which formed a covalent bond in polymers [6]. Previous work has shown that CONH_2_–functionalized carbon nanotubes have the best enhancement effect when the degree of functionalization is 10% [7]. It has been proved that the mechanical properties of pure NBR show a decline as temperature increases [8,9], which can be enhanced by the addition of PCNT at both low and high temperatures [10]. Obviously, further research of the reinforcement at different temperatures for the functionalized CNT needs to be conducted.

Molecular dynamics (MDs) simulation has emerged as an alternative method to study the properties of material at the atomic scale by providing microscopic detailed information of molecular interactions. It is capable for further interpretations of experimental results, which is also effective in estimating the mechanical properties of materials. Based on molecular dynamics theory, this paper uses CNT as nano-reinforcing materials, –CONH_2_ functional groups to modify CNT. The functionalized carbon CONH_2_–CNT/NBR matrix is simulated and calculated by NBR as the polymer matrix, the nanomaterial enhances the mechanical properties of the NBR matrix [11]. The difference of performance between functionalized carbon nanomaterials and the NBR matrix at different temperatures was compared and analyzed. The main analysis focused on mechanical properties, free volume fraction, MSD and diffusion coefficient, hydrogen bond, dipole autocorrelation function, and the impact of performance and unplugging behavior, provided a theoretical basis for the structural design of high–performance NBR–based nanocomposites [12].

## 2. Modeling and Methods

**Figure 1:**
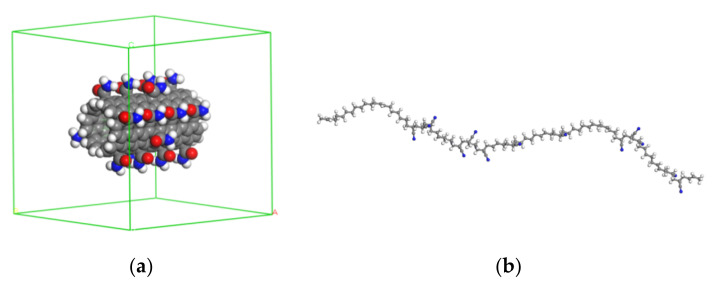

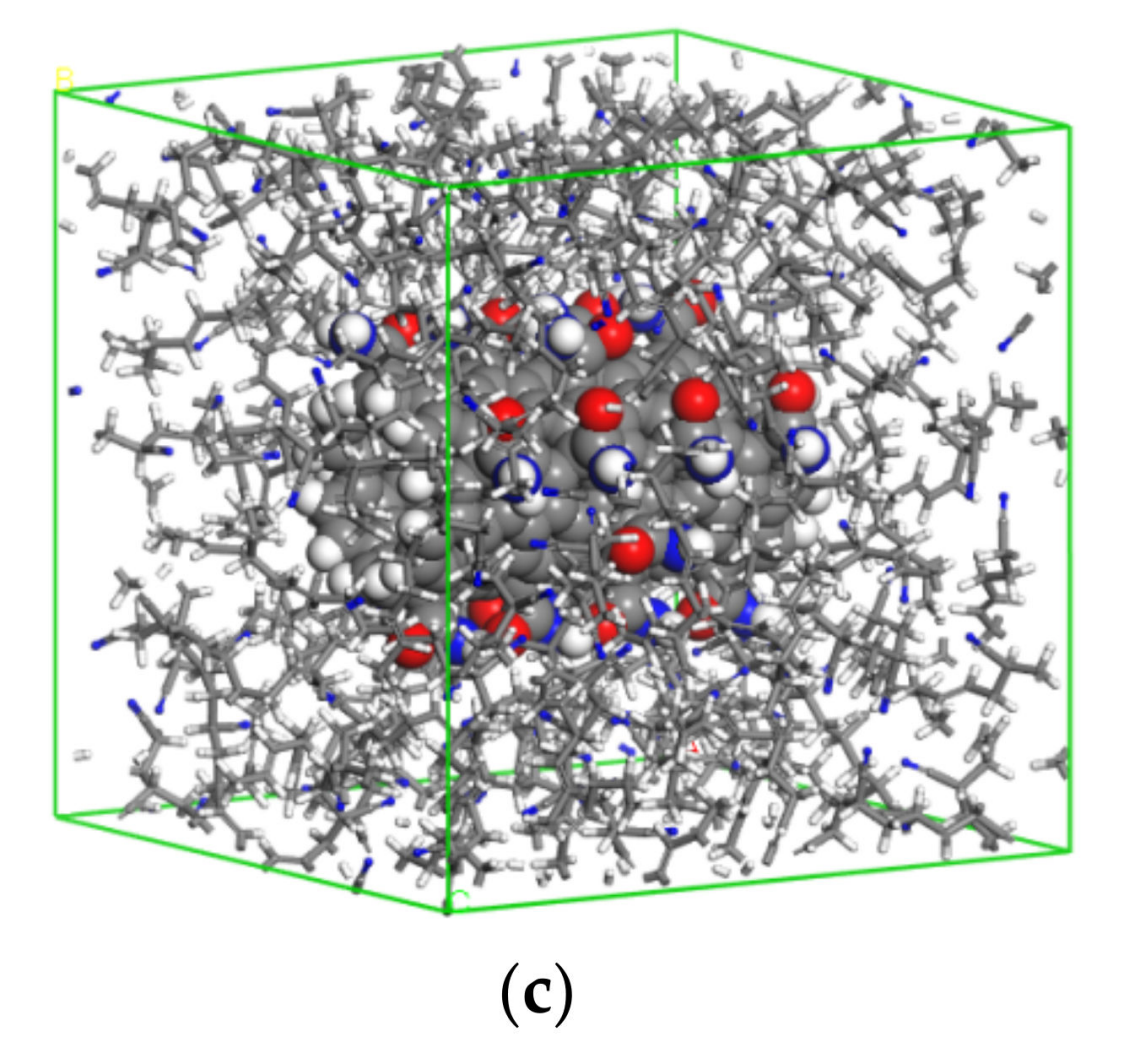
Molecular models: (**a**) an amide-functionalized CNT, (**b**) a single chain of NBR, (**c**) amide–functionalized CNT/NBR composite.

To investigate the properties of the composites at different temperatures, a pure CNT/NBR composite and a composite with amide groups models were established, respectively. The specific model construction method is as follows: carbon nanotubes (CNT) and nitrile rubber (NBR) are used as representatives of reinforcing materials and polymer matrix, and amorphous molecular models of CNT and NBR matrix are established, respectively. First, two (6,6) armchair-shaped single-walled carbon nanotubes are constructed with a length of 29.51 Å and a diameter of 8.0 Å, both ends of which are terminated with hydrogen atoms, and then two of them are placed, each with a size of 50 × 40 × 40 nm^3^ periodic lattice center, for functionalized CNT, amide group (CONH_2_), and about 10% functionalization degree is uniformly grafted on the sidewall of CNT, as shown in Figure 1a.

As shown in Figure 1b, acrylonitrile (C_3_H_3_N) and 1,3–butadiene (CH_2_=CH–CH=CH_2_) were employed as repeating units to build an NBR molecular chain with a chain length of 20. As shown in Figure 1c, the NBR molecular chains were randomly encapsulated into periodic structural units containing CNT according to the set density of 1.00 g/cm^3^.

To obtain the global and local minimum energy configuration, the above-mentioned molecular models were successively subjected to the process of geometric optimization and molecular dynamics equilibration. The conjugate gradient method with an energy convergence accuracy of 10^−5^ kcal/mol is used to optimize the geometry of the composite material system. The molecular dynamics equilibration of the Normal Canonical ensemble (NVT) system was run at different temperatures for 600 ps to eliminate the internal stress of the molecular system, e.g., −20 °C, 20 °C, 60 °C, 100 °C, 140 °C, respectively. Molecular dynamics simulations of the NVT molecular system were performed at 600 ps under the conditions of constant pressure (101 Kpa) and the above five different temperatures. Taking 140 °C as an example, the system configuration after complete equilibration was obtained, and the properties of the composites were calculated according to the final trajectory frame of 80 ps. The simulation process of the constant strain method is to apply 4-times strain ε (−0.003, −0.001, 0.001, 0.003) in the six directions of x, y, z, xy, xz, and yz, respectively.

## 3. Results and Discussions

### 3.1. Mechanical Properties of the Composites at Different Temperatures

**Figure 2:**
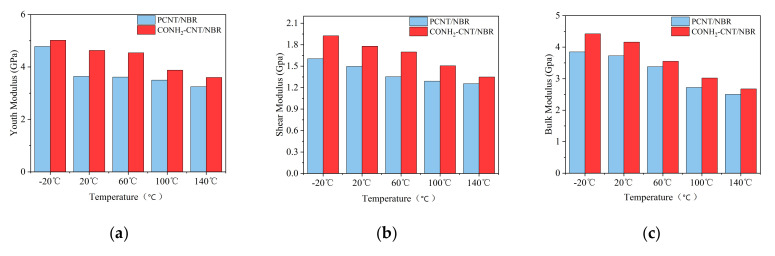
Mechanical properties of composites. (**a**) Young’s modulus, (**b**) shear modulus, (**c**) bulk modulus.

Young’s modulus, shear modulus, and bulk modulus are parameters of mechanical properties of the material, which reveal the stiffness, shear resistance, and resistance of volume change, respectively. To study the mechanical properties of the CONH_2_–CNT/NBR composite and PCNT/NBR composite at different temperatures, the reinforcement differences at five different temperatures of −20 °C, 20 °C, 60 °C, 100 °C, and 140 °C were compared and analyzed with the strain constant method by MD simulations. To obtain the mechanical properties of the NBR model, stretching operations were performed using MDs simulations. The tensile process was conducted with the uniform change of the shape of the simulation box during the MDs simulations, after applying a pressure, mechanical properties can be calculated using the virial formula. The calculation results are shown in Figure 2.

Due to the anisotropy of the composites, Young’s modulus value takes the average value in the X, Y, and Z directions. As shown in Figure 2, under different condition temperatures of −20 °C, 20 °C, 60 °C, 100 °C, and 140 °C, the Young’s modulus of PCNT/NBR composite was 4.78, 3.64, 3.61, 3.50, and 3.25 Gpa, respectively. In addition, the Young’s modulus of the CONH_2_–CNT/NBR material at the same temperature was 5.02, 4.63, 4.54, 3.88, and 3.60 Gpa, respectively. The result showed that, compared with PCNT/NBR material, the Young’s modulus of CONH_2_–CNT/NBR composite at different temperatures increased by 5.02%, 27.2%, 25.8%, 10.86%, and 10.77%, respectively, higher stiffness of the composite was obtained. Under the same temperature conditions as above, the shear modulus of PCNT/NBR composite was 1.60, 1.50, 1.35, 1.29, and 1.26 Gpa, respectively, while that of the CONH_2_–CNT/NBR composite was 1.93, 1.78, 1.70, 1.51, and 1.35 Gpa, respectively. The shear modulus of CONH_2_–CNT/NBR composite at the same temperature has increased by 20.63%, 18.67%, 25.93%, 17.05%, and 7.14%, respectively, an obvious shear resistance had been achieved. Moreover, the bulk modulus of NBR composite enhanced with PCNT/NBR were 3.85, 3.72, 3.38, 2.72, and 2.50 GPa, in the case of CONH_2_–CNT, they were 4.42, 4.16, 3.55, 3.02, and 2.68 GPa. Compared with the bulk modulus of the PCNT/NBR composite, the bulk modulus of the CONH_2_–CNT/NBR composite increased by 14.81%, 11.83%, 5.03%, 11.03%, and 7.20%, respectively, leading to a better resistance of volume change.

In summary, the functionalization of CNT is beneficial to improve the mechanical properties of NBR composites, including strength and stiffness [13,14], and the mechanical properties of the CONH_2_–CNT/NBR composite are better than that of PCNT/NBR at the same temperature. This superiority exists in both the high and low temperature ranges.

### 3.2. Fractional Free Volumes of the Composites at Different Temperatures

FFV, which reflects the tightness and compatibility for polymer nanocomposites, was calculated to investigate the tightness and compatibility of CNTs and NBR; the lower the FFV was, the higher the tightness and the better the compatibility of the composite. To explore the mechanism of mechanical properties’ enhancement of composites, the motion of molecular chains should be analyzed. For verification of the influence of the above temperature on the consistency of the composite, the fractional free volume (FFV) of the PCNT/NBR material and CONH_2_–CNT/NBR composite were calculated based on the NVT molecular dynamics equilibration trajectory file.

**Figure 3:**
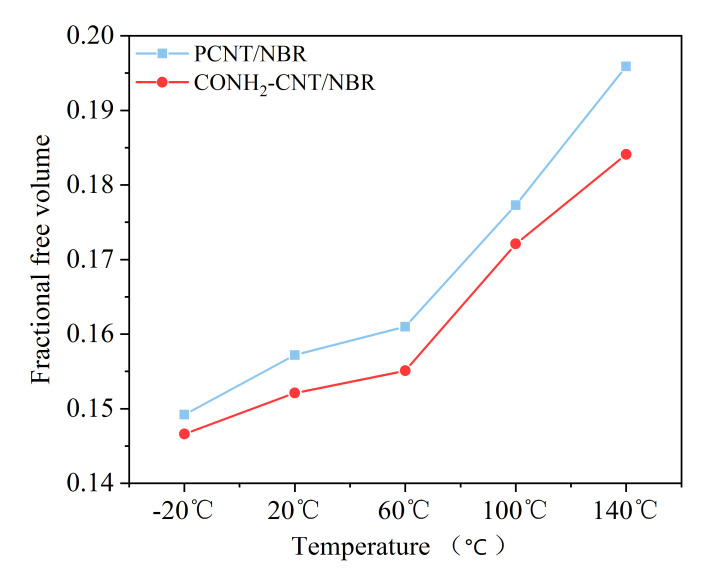
Fractional free volumes of composites during MD simulations.

As shown in Figure 3, as the temperature increases, the fractional free volume values of PCNT/NBR composites were 14.92%, 15.72%, 16.10%, 17.73%, and 19.59%, respectively, and in the case of CONH_2_–CNT/NBR, 14.66%, 15.21%, 15.51%, 17.21%, and 18.41%, respectively. The material becomes loose with the temperature increase. Meanwhile, at the same temperature, the fractional free volume of the functionalized composite was lower than that of the PCNT/NBR material.

The functionalization of CNT is beneficial to inhibit the influence of temperature on the FFV of the NBR matrix from the analysis results, which can further compress the free movement space of the NBR molecular chain [15]. This is due to the higher interface adsorption capability of CONH_2_–CNT/NBR composite. Therefore, more NBR molecular chains are adsorbed on the surface of functionalized CNT, thereby forming a more stable NBR matrix and further inhibiting the decrease of mechanical properties.

### 3.3. MSDs of the Composites at Different Temperatures

**Figure 4:**
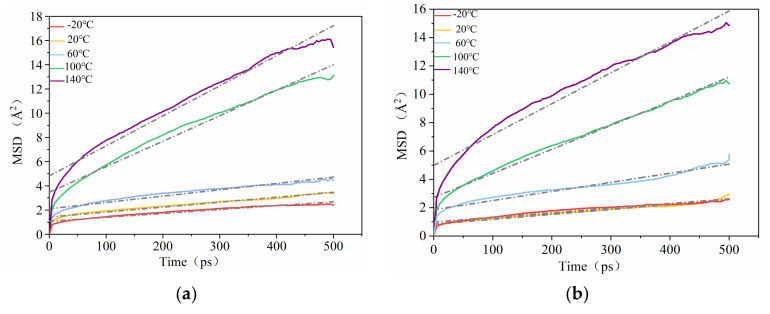
MSDs curves of NBR chains during MD simulations. (**a**) PCNT/NBR composite, (**b**) CONH_2_–CNT/NBR composite.

For further evidence that the functionalization of CNT may further restrict the molecular chains from moving, the MSD of the PCNT/NBR material and CONH_2_–CNT/NBR composite at five different temperatures including –20 °C, 20 °C, 60 °C, 100 °C, and 140 °C was calculated based on the NPT molecular dynamics equilibration trajectory. The mean square displacement (MSD) of the PCNT/NBR material and CONH_2_–CNT/NBR composite are shown in Figure 4. It can be seen that the average mean square displacement values of the NBR molecular chains of the PCNT/NBR composite at different temperatures were 1.80, 1.72, 3.48, 6.96, and 10.42 Å^2^, respectively, while the MSD of the CONH_2_–CNT/NBR molecular chains were 1.48, 2.36, 3.49, 5.67, and 7.72 Å^2^, respectively. As the temperature increased, the MSD of both of the composites increased, and by comparing them, the average mean square displacement value of the PCNT/NBR molecular chain was slightly lower than that of the CONH_2_–CNT/NBR one. The main reason for this result is the acceleration of molecular motion at high temperatures. However, due to the existence of hydrogen bonds in the CONH_2_–CNT/NBR composite, the molecular motion is restricted. MSDs of NBR composites are decreased by the addition of the functionalized CNTs at each temperature. Consequently, a smaller free space and more stable structure of the composites are obtained. It can be concluded that better dispersibility [16], stability, and stronger interfacial interactions are formed between the CONH_2_–CNT/NBR matrices than PCNT/NBR at each temperature, which renders the structure of NBR composites denser and hinders the movement of NBR chains. The above conclusions can be confined that at high temperatures, hydrogen bonds play a positive role in the stability of the composite [17].

### 3.4. Simulation of the Pull-Out Behavior and Interfacial Binding Energy of the Composites in Different Temperatures

**Figure 5:**
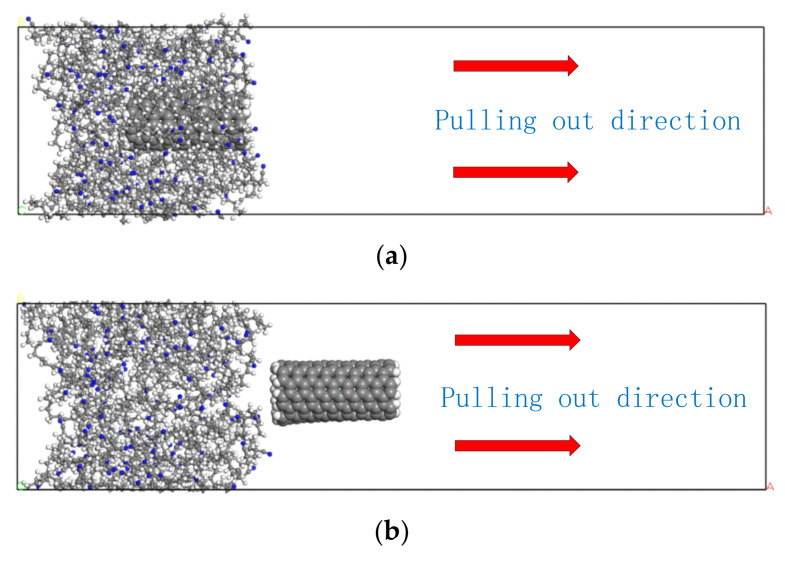
Sketch of pull–out structure. (**a**) Before pulling out, (**b**) after pulling out.

To explore the inherent mechanisms of various amido–amine functionalized CNTs on the mechanical properties of NBR composites at different temperatures, the variation of interfacial binding energies between the functionalized CNTs and NBR matrices with the increase temperature were compared and analyzed by pulling out simulation. Five different temperature parameters were again set at −20 °C, 20 °C, 60 °C, 100 °C, and 140 °C, for CNT to perform a molecular dynamics simulation of pulling out from the NBR matrix. Therefore, after the optimal molecular configuration of the composite is obtained, the initial speed of PCNT and CONH_2_–CNT in the horizontal direction was 15 Å/ps, which allows the reinforcing material to slide relatively through the NBR matrix, and this was performed at different temperatures, respectively. They were continuously pulled out from the NBR matrix. It should be pointed out that in the molecular dynamics simulation process, the NVE ensemble is used to maintain the conservation of total energy [18]. The sliding distance and speed changes of PCNT and CONH_2_–CNT are calculated and recorded respectively. Snapshots of PCNT and CONH_2_–CNT pullout simulations at different temperatures are shown in Figure 5: as the temperature increases, the interface effect gradually weakens.

**Figure 6:**
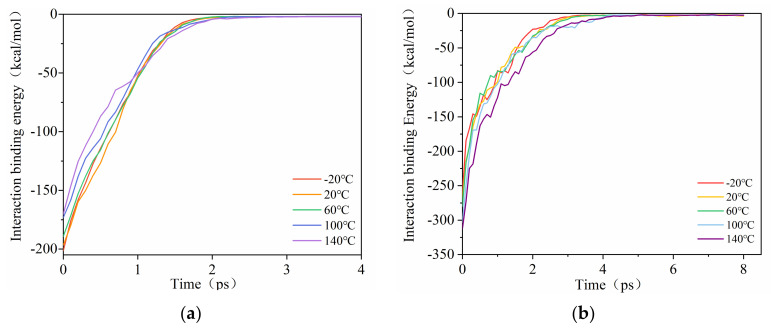
Variations of the interfacial potential energy between the CNT and NBR matrices during the pull–out process. (**a**) PCNT/NBR composite, (**b**) CONH_2_–CNT/NBR composite.

To further reveal the microscopic mechanism of the difference in the interface interaction between PCNT and CONH_2_–CNT and the rubber matrix, the changing trend of the interfacial binding energy between PCNT and CONH_2_–CNT and the NBR matrix during the pull-out simulation process was calculated and extracted. As shown in Figure 6, at different temperatures, the average interfacial binding energie between CNT and the rubber matrix before pulling-out was −201.30, −196.50, −189.10, −173.07 and −170.20 kcal/mol, respectively. The average interfacial binding energy between CONH_2_–CNT and the rubber matrix was −259.50, −269.41, −279.72, −303.21, −311.92 kcal/mol, respectively. The CNT functional group also played a certain role in the pull−out simulation, and CONH_2_–CNT hindered the polymer chains from moving, the effect was more obvious at high temperatures [19].

**Figure 7:**
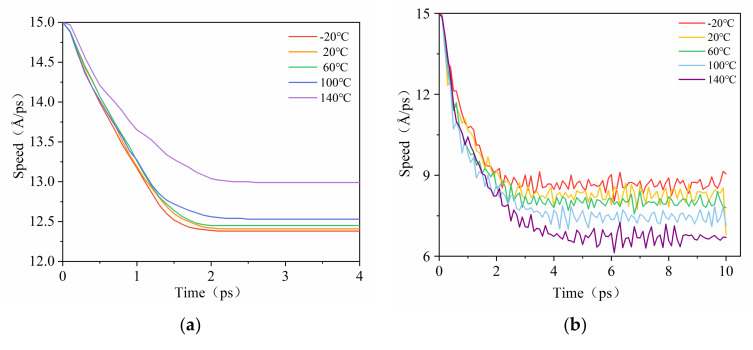
The variations of the sliding velocities of the CNT during the pull–out process. (**a**) PCNT/NBR composite, (**b**) CONH_2_–CNT/NBR composite.

As shown in Figure 7, the sliding velocities of the CNT during the pull-out process decreased after the functionalization of CNT in each temperature, which also revealed a grow of the resistance. Therefore, through the above discussion of the interfacial binding energy, it can be concluded that as the temperature increases, the interfacial binding energy between PCNT and NBR decreased to a lower degree and that between PCNT and CONH_2_–CNT growed, so due to the bridging effect during the crack propagation process, delaying crack growth was inhibited to increase the service life of rubber, CONH_2_–CNT has a more obvious effect than PCNT both at low and high temperatures [20].

To explore the inherent mechanisms of CNT reinforcement on the mechanical properties of NBR, the contribution of nonbonding energy terms to the interfacial binding energy was calculated. As shown in Table 1 and Table 2, according to the comparison of the two kinds of composites, electrostatic force played a more important role in the CONH_2_–CNT/NBR composite than in the PCNT/NBR composite. Meanwhile, with the temperature increasing, the portion of electrostatic force in the interfacial binding energy increased. This phenomenon is supposed to be attributed to the hydrogen bond interaction between CONH_2_–CNT and NBR, thus making CONH_2_–CNT/NBR composites have higher resistance to external forces.

### 3.5. The Number of Hydrogen Bonds at Different Temperatures

**Figure 8:**
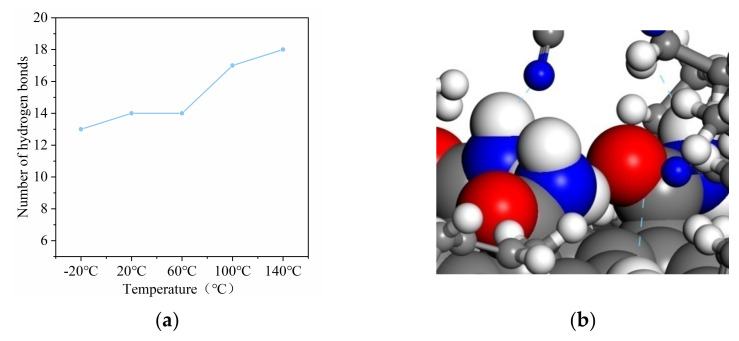
NHB between the CONH_2_–CNTs and NBR matrices (**a**) Variations of NHB, (**b**) snapshot of NHB.

For further verification of the conjecture in 3.4, the influence of different temperatures in the equilibration trajectory of NVT molecular dynamics on the change of the number of hydrogen bonds between the CONH_2_–CNT and NBR matrix was also calculated. As shown in Figure 8, in the range of −20–140 °C, the average NHB of CONH_2_–CNT/NBR composite was 13, 14, 14, 17, and 18, respectively. The results show that with the increase of temperature, the NHB of the CONH_2_–CNT/NBR composite grows, which further contributes to the restriction of the motion of molecular chains, and the better performance than the PCNT/NBR material. The hydrogen bonds formed by the functionalization of CNT has played a more—and—more important role in the optimization of the composite as temperature increases. Since chemical bonds contribute to further enhancement of the material, other modification methods such as cross-linking of the NBR by amino may be taken into account and compared in subsequent research.

### 3.6. Dipole Autocorrelation Function of Composites at Different Temperatures

**Figure 9:**
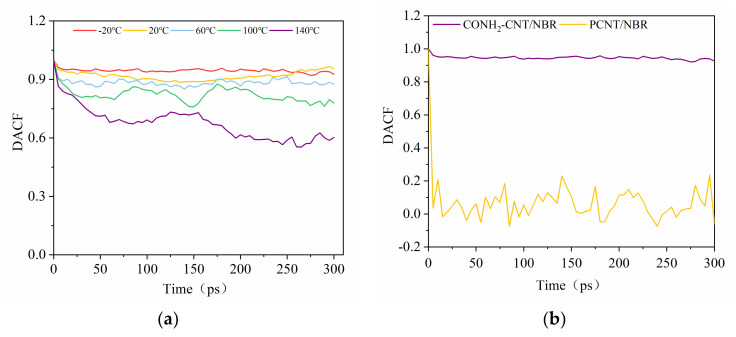
The DACFs of the composites. (**a**) Variation of DACFs of CONH_2_–CNT/NBR at different temperatures, (**b**) comparison between CONH_2_–CNT/NBR and PCNT/NBR at 20 °C.

Based on the micromechanics, the overall performances of the composite at the nanoscale at different temperatures are relative to the characteristics of the matrix, modification of CNT, and their interfaces. To explore the variation of the interfacial interaction mechanism at each temperature, the dipole autocorrelation function (DACF) of CNTs and were determined, and that of the composites at different temperatures in the molecular dynamics equilibration trajectory of NVT was calculated. Taking 20 °C for example, it is indicated that stronger dipole—dipole interactions are formed in the CONH_2_–CNT/NBR composite than that in the PCNT/NBR composite, which greatly limits the motions of polymer chains in the CONH_2_–CNT/matrix. Meanwhile, the fluctuation amplitudes of DACFs reveal the vibration behavior of the polymer, as shown in Figure 9a, and the value of DACF at each temperature remains consistent, making the functionalized NBR more stable in the matrix, reducing agglomeration, and it has better dispersibility in the matrix than the PCNT/NBR composite [17]. With the increase of temperature, dipole—dipole interactions from–CONH_2_ polar amide functional groups, hydrogen bonding interactions between amide groups on the surface of CNTs and polar nitrile groups of NBR matrices, and interfacial interactions between the CONH_2_–CNT and NBR matrix, remain relatively consistent, making the composite obtain a higher resistance to external forces at each temperature.

## 4. Conclusions

MDs simulations were performed at different temperatures to study the mechanical properties of PCNT–/NBR and CONH_2_–CNT/NBR composites, and the mechanisms according to their FFVs, MSDs DACFs, and NHS. Conclusions are drawn as below:

(1) Five different temperatures and two different models, including pristine carbon nanotubes and functionalized carbon nanotubes with polar functional group, CONH_2_, were used to study the properties of the composite in different temperatures. Comparing the results of the mechanical properties of the PCNT/NBR composite and the CONH_2_–CNT/NBR material with different temperatures, the results show that the Young’s modulus, shear modulus, and bulk modulus of the two materials are reduced, but the CONH_2_–CNT/NBR composite performs better at each temperature with an improvement of the modulus by 5.02–25.93%.
(2) The functionalization of CNT is beneficial to inhibit the influence of temperature on the FFV of the NBR matrix, it can further compress the free movement space of the NBR molecular chain in high temperatures. Therefore, more NBR molecular chains are adsorbed on the surface of functionalized CNT, the molecular motion is restricted. MSDs of the NBR composite are clearly decreased by the addition of the functionalized CNTs, thereby forming a more stable NBR matrix and further inhibiting the decrease of mechanical properties in high temperatures.
(3) Interfacial binding energy is the fundamental reason for the enhancement of CNT on NBR. According to the comparison between the pull-out behaviors of two kinds of composite, electrostatic force played a more important role in the CONH_2_–CNT/NBR composite than in the PCNT/NBR composite, especially in high temperatures. This is attributed to the hydrogen bond interaction between CONH_2_–CNT and NBR which was increased as the temperature increased, having a bridging effect during the crack propagation process. Meanwhile, the value of DACF at each temperature remains consistent, making the functionalized NBR more stable in the matrix at both low and high temperatures, reducing agglomeration, and it has better dispersibility in the matrix than PCNT/NBR composite.

## Figures and Tables

**Table 1:** Contribution of nonbonding energy terms to interfacial binding energy in CONH_2_–CNT/NBR composite.

Temperature	U*_inter_* (kcal/mol)	U*_elec_*	Portion of U_elec_	U*_vdW_*	Portion of U_vdW_
−20 °C	−201.30	−9.63	4.79%	−191.67	95.21%
20 °C	−196.50	−11.24	5.72%	−185.27	94.28%
60 °C	−189.10	−2.51	1.33%	−186.58	98.67%
100 °C	−173.07	−8.75	5.05%	−164.32	94.95%
140 °C	−170.20	−3.90	2.29%	−166.29	97.71%

**Table 2:** Contribution of nonbonding energy terms to the interfacial binding energy in PCNT/NBR composite.

Temperature	U*_inter_* (kcal/mol)	U*_elec_*	Portion of U_elec_	U*_vdW_*	Portion of U_vdW_
−20 °C	−259.50	−66.57	25.65%	−192.93	74.35%
20 °C	−269.41	−68.90	25.58%	−200.50	74.42%
60 °C	−279.72	−90.28	32.27%	−189.44	67.73%
100 °C	−303.21	−98.92	32.62%	−204.29	67.38%
140 °C	−311.92	−113.01	36.23%	−198.91	63.77%

## Data Availability

Not applicable.

## References

[1] Kim H.J., Kim D.E. (2012). Molecular dynamics simulation of atomic-scale frictional behavior of corrugated nano-structured surfaces. Nanoscale.

[2] Wang X., Chen D.L., Zhong W.S., Zhang L., Fan X.Q., Cai Z.B., Zhu M.H. (2020). Experimental and theoretical evaluations of the interfacial interaction between carbon nanotubes and carboxylated butadiene nitrile rubber: Mechanical and damping properties. Mater. Des..

[3] Li Q.Y., Dong Y.L., Perez D., Martini A., Carpick R.W. (2011). Speed Dependence of Atomic Stick-Slip Friction in Optimally Matched Experiments and Molecular Dynamics Simulations. Phys. Rev. Lett..

[4] Palaty S., Joseph R. (2006). Low Temperature Curing of NBR for Property Improvement. J. Elastomers Plast..

[5] Yang B., Wang S.J., Li Y.L., Li T., En H., Song S.Y. (2020). Molecular dynamics simulations of mechanical properties of swollen nitrile rubber composites by incorporating carbon nanotubes. Polym. Compos..

[6] Chakraborty S.K.M.K.K. (1993). Studies on Curing Characteristics of Natural Rubber-, Nitrile Rubber and Silicone Rubber-Based Gum Vulcanizates in the Presence of Boron Compounds. J. Elastomers Plast..

[7] Cui J.Z., Zhao J., Wang S.J., Wang Y., Li Y.L. (2021). Effects of carbon nanotubes functionalization on mechanical and tribological properties of nitrile rubber nanocomposites: Molecular dynamics simulations. Comput. Mater. Sci..

[8] Luo W., Li M., Huang Y., Yin B., Hu X. (2019). Effect of temperature on the tear fracture and fatigue life of carbon-black-filled rubber. Polymers.

[9] Schieppati J., Schrittesser B., Wondracek A., Robin S., Holzner A., Pinter G. (2021). Temperature impact on the mechanical and fatigue behavior of a non-crystallizing rubber. Int. J. Fatigue.

[10] Ata S., Tomonoh S., Yamda T., Hata K. (2017). Improvement in thermal durability of fluorinated rubber by the addition of single-walled carbon nanotubes as a thermally stable radical scavenger. Polymer.

[11] Zheng G.B., Sano H., Nakagoe O., Tanabe S.J. (2018). A Molecular Dynamics Simulation of the Tensile Behavior of Y-B ranched-CNT/SiC Nanocomposite. Key Eng. Mater. Submitt..

[12] Fan D., Yang S.T., Saafic M. (2021). Molecular dynamics simulation of mechanical properties of intercalated GO/C-S-H nanocomposites. Comput. Mater. Sci..

[13] Abdul R.E.S., Muhd J.N.B., Abdul H.Y.W. (2018). Reinforcement effect of nanocellulose on thermal stability of nitrile butadiene rubber (NBR) composites. J. Appl. Polym. Sci..

[14] Lev Y., Faye A., Volokh K.Y. (2018). Experimental Study of the Effffect of Temperature on Strength and Extensibility of Rubberlike Materials. Exp. Mech..

[15] Lee J.K., Park J., Kang Y.C., Koh S.W. (2016). Spectroscopic and Mechanical Properties of Nano Silica Rubber Composite Material. J. Chosun Nat. Sci..

[16] Alian A.R., Meguid S.A. (2017). Molecular dynamics simulations of the effect of waviness and agglomeration of CNTs on interface strength of thermoset nanocomposites. Phys. Chem. Chem. Phys..

[17] Li Y., Wang S., Arash B., Wang Q. (2016). A study on tribology of nitrile-butadiene rubber composites by incorporation of carbon nanotubes, Molecular dynamics simulations. Carbon.

[18] Varghese T.V., Kumar H.A., Anitha S., Rajeevc R.S., Rao V.L. (2013). Reinforcement of acrylonitrile butadiene rubber using pristine few layer graphene and its hybrid fillers. Carbon.

[19] Yang B., Wang S., Song Z., Liu L.F., Li H.L., Li Y.L. (2021). Molecular dynamics study on the reinforcing effect of incorporation of graphene/carbon nanotubes on the mechanical properties of swelling rubber. Polym. Test..

[20] Liu J., Li X., Xu L., Zhang P.Q. (2016). Investigation of aging behavior and mechanism of nitrile-butadiene rubber (NBR) in the accelerated thermal aging environment. Polym. Test..

